# Assessing the Relative Contribution of DSB Repair Proteins as a Function of LET

**DOI:** 10.1016/j.ijpt.2025.101198

**Published:** 2025-07-26

**Authors:** Francisco D.C. Guerra Liberal, Shannon J. Thompson, Lydia L. Gardner, Jason L. Parsons, François Chevalier, Kevin Tabury, Stephen J. McMahon

**Affiliations:** 1 The Patrick G Johnston Center for Cancer Research, Queen's University Belfast, Belfast, United Kingdom; 2 Institute of Cancer and Genomic Sciences, University of Birmingham, Birmingham, United Kingdom; 3 UMR6252 CIMAP, CEA-CNRS-ENSICAEN, Normandie Université, Team Applications in Radiobiology with Accelerated Ions, 14000 Caen, France; 4 Radiobiology Unit, Nuclear Medical Applications, Belgian Nuclear Research Center, Mol, Belgium

**Keywords:** Relative biological effectiveness, Linear energy transfer, Proton therapy, Carbon ions, Damage repair

## Abstract

**Purpose:**

Particle therapy is gaining popularity due to its dosimetric benefits. Particle radiation also has a higher linear energy transfer (LET) than X-rays, leading to more complex DNA damage and a higher relative biological effectiveness (RBE). While potentially beneficial, there remains significant uncertainty in how RBE depends on genetic features of irradiated cells. Understanding how cells respond to and repair these damages is crucial for optimising radiotherapy.

**Materials and Methods:**

This study evaluates how loss of different DNA double strand break (DSB) repair genes impacts on radiosensitivity. CRISPR-modified RPE-1 cells were exposed to 6 different LETs using X-rays, protons, carbon ions, and alpha particles, following which clonogenic survival and DNA DSB repair kinetics were measured. Experimental data were then compared with predictions from a mechanistic model of radiation response (Medras).

**Results:**

Clonogenic assays showed that cells lacking ATM and NHEJ repair genes were particularly radiosensitive, even for high LET exposures. While RBE increased with LET for all analysed knockout lines, RBE increased at a slower rate for cells that were more sensitive to X-rays, regardless of the affected pathway. Moreover, data showed no significant difference in DNA repair pathway dependence as a function of LET. Medras-predicted responses were in good agreement with both the genetic background and LET dependencies of radiosensitivity, without any assumption of a change in repair pathway dependence with LET.

**Conclusion:**

This research further highlights the importance of DSB repair pathways, particularly NHEJ, in determining cellular sensitivity to different radiation qualities, but suggests that in this system there is little difference in pathway dependence between X-rays and high-LET radiation. Mechanistic approaches like Medras offer a promising approach to predict radiation responses, to support more personalised and effective cancer treatments based on genetic profiles.

## Introduction

Radiotherapy is a cornerstone of cancer treatment, used for approximately half of all cancer patients, and standing as the second most curative approach, following surgery.^1^ Traditionally, radiotherapy has employed high-energy X-rays, but particle radiotherapy, utilising protons and carbon ions, has gained increasing traction. In 2023, 117 clinical facilities globally were offering particle radiotherapy. The primary advantage of particle radiotherapy lies in its dosimetric benefits. Unlike X-rays, charged particles deposit the majority of their energy at the end of their trajectory, a phenomenon known as the Bragg peak. This allows for precise targeting of tumours, reducing side effects by sparing surrounding healthy tissues.^2^

Charged particles also induce more complex DNA damage than X-rays, due to their higher linear energy transfer (LET). This results in a higher relative biological effectiveness (RBE). Clinical proton beams have LETs of approximately 2 to 3 keV/μm in the middle of the spread-out Bragg Peak and can reach 10 to 15 keV/μm at the distal end.^3^ Clinical carbon ion beams have an average LET of 40 to 80 keV/μm within the spread-out Bragg Peak, escalating to around 200 keV/μm at the distal end.^4^ For context, a clinical 6 MV X-ray beam has an LET of approximately 0.2 keV/μm.^5^, ^6^

These LET variations, along with differences in particle energy and charge, result in distinct ionisation patterns and radiobiological effects at molecular and cellular levels. The RBE of a given charged particle increases rapidly with increasing LET, reaching a peak at around 100 to 200 keV/μm, after which it decreases due to a saturation effect known as "overkilling."^7^

Complex DNA damage from high LET radiation has been suggested to be notably harder for cells to repair than simpler X-ray lesions.^8^ DNA double strand breaks (DSBs) are critical as their repair, or lack thereof, can lead to genetic mutations, carcinogenesis, and cell death, making cellular DSB repair capacity a crucial determinant of radiation response. Hence, understanding differential cellular responses to high LET-induced DNA damage is imperative to optimise tumour control and mitigate side effects.

While mammalian cells are proficient in repairing DSBs, studies using γH2AX foci have shown that repair rates decrease and residual DNA breaks increase with higher-LET radiation.^9^ Historically, it was believed that DNA repair played a reduced role following high-LET radiation, implying particle radiotherapy might be more effective in tumours with robust repair mechanisms.^10^ However, recent research highlighted the significance of functional DNA repair even after high-LET radiation.^11^, ^12^ Understanding how different radiation qualities interact with DNA repair pathways is crucial for optimising therapeutic outcomes.

Cells repair DSBs through 3 key pathways: nonhomologous end joining (NHEJ), homologous recombination (HR), and alternative end joining (alt-EJ). Each pathway plays a distinct role in responding to radiation-induced damage: NHEJ is the predominant repair mechanism in mammalian cells, operating throughout the cell cycle. This pathway directly ligates DNA ends without requiring sequence homology, making it a rapid but error-prone process. NHEJ is particularly effective for repairing relatively sparse DSBs caused by low doses of low-LET radiation. However, high-dose or high-LET radiation can generate complex, clustered DSBs, which can overwhelm the NHEJ machinery leading to high rates of misrepair.^13^ HR is a high-fidelity repair mechanism that uses a homologous sequence as a template, only available during the late S and G2 phases of the cell cycle. HR is associated with improved cellular survival and is suggested to be better equipped to resolve complex DSBs. Finally, Alt-EJ serves as a backup repair pathway when NHEJ is impaired or fails. This mechanism relies on regions of microhomology for end rejoining, making it highly error-prone and prone to introducing deletions and chromosomal rearrangements. High-LET radiation is thought to enhance the utilisation of alt-EJ due to the inability of NHEJ to effectively process complex damage.

It has been suggested that complex DNA breaks induced by high-LET radiation are preferentially repaired through the HR pathway. This has been supported by studies that indicated a higher dependency on HR repair following high-LET exposure compared to low-LET exposure.^14^, ^15^, ^16^, ^17^ Conversely, some research has not found a strong correlation between HR capacity and RBE, asserting that classic NHEJ remains the primary DSB repair mechanism.^18^, ^19^, ^20^ Loss of NHEJ appears to have a more substantial radiosensitizing effect than loss of HR, even with carbon ions.^21^, ^22^ However, it has recently been highlighted that the increased RBE associated with particle radiation likely stems from the distinct nanoscale dose distribution patterns that may favour the alt-EJ pathway,^23^ which induces significant deletions and chromosomal rearrangements, as observed in chromosomal aberration studies.^24^

Genetic and biological differences among cancers significantly influence radiosensitivity, affecting clinical outcomes.^25^ Despite this, the application of radiosensitivity models to personalise radiotherapy based on genetic or biological differences has been limited due to challenges in accurately predicting radiation responses. Thus, improving our understanding of biological responses to different forms of radiotherapy, particularly photons and particle therapy, is crucial.

This study evaluates the relative importance of various DSB repair pathways in normal cells following low- and high-LET exposures, to explore the potential effects of differences in DSB complexity. This study combines experimental data at 6 different LETs using 4 different radiation types with a mechanistic model of radiation response (Medras)^26^ to investigate the impact of dysregulation of DNA repair pathways on RBE and overall radiosensitivity. Enhanced knowledge of cellular and molecular responses to low- and high-LET radiation will inform the design of future clinical trials and potentially support personalised patient allocation for particle radiotherapy based on their cancer’s genetic alterations.

## Material and methods

### Cell lines

RPE-1 cells, acquired from ATCC, were cultured in DMEM/F-12 with L-glutamine, 15 mM HEPES, 10% FBS, and 1% penicillin-streptomycin. The cells were maintained at 37°C in a 5% CO_2_ atmosphere. CRISPR/Cas9 knockout was performed on these cells targeting the genes P53, ATM, DCLRE1C (Artemis), BRCA1, LIG4, and PRKDC (DNA-PKcs), and validated, as previously described.^27^ Single clones with null expression of the protein of interest were selected for this study.

### Irradiation setups

X-ray irradiation was performed using an X-RAD 225 radiation source (Precision X-ray Inc, USA) at Queen’s University Belfast, operating at 225 kV with a 2 mm copper filter and a current of 13.3 mA. This setup yielded a dose rate of 0.59 Gy/min at a distance of 50 cm from the source. Cells were exposed to doses of 0.6, 1, 2, 4, 6, and 8 Gy as previously described.^27^ Monte Carlo simulations of the secondary electron spectrum of the 225 kVp X-ray cabinet used in this study give an LET of 0.45 keV/μm.^28^

Proton irradiations were conducted at the Douglas Cyclotron at the Clatterbridge Cancer Centre. Proton irradiation setup and conditions were previously described.^20^ In short, for high-energy protons, cells were irradiated directly using a 1 keV/μm pristine beam with an effective energy of 58 MeV and a dose rate of ∼5 Gy/min. Low-energy proton irradiations were performed using a modulator to generate a 27 mm spread-out Bragg peak. A 24.4 mm absorber positioned the cells at the distal edge, corresponding to a mean proton energy of 11 MeV and a dose-average LET of 12 keV/μm, with a dose rate of ∼5 Gy/min.

Carbon ion exposures were conducted at the iRiA platform of CIRIL-CIMAP (GANIL, Caen, France) using carbon ions with an energy of 95 MeV/A and an LET of 28 keV/μm.^29^ Irradiations were performed at a dose rate of approximately 1 Gy/min, positioning cells in the plateau region of the Bragg curve to ensure consistent LET throughout the cell thickness. To achieve the desired LET at the cell layer, the carbon ion beam energy was adjusted from the native level to 71 and 26 MeV/A using polymethyl methacrylate (PMMA) beam degraders of 6.9 and 16.9 mm thickness, respectively. This resulted in LET values in water of 34 and 73 keV/µm. The fluence of carbon ions (P/cm²) was used to determine the absorbed radiation dose (Gy). Flasks were irradiated vertically, completely filled with medium, which was promptly replaced after irradiation.

For alpha irradiation, circular Mylar dishes with a thickness of 0.9 µm and a surface area of 9.1 cm² were positioned 2.9 mm from an uncollimated ^241^Am alpha-source, with a dose rate of 1.57 Gy/min, at Queen’s University Belfast. The average energy at the cell layer was 2.88 ± 1.04 MeV with an LET of 129.3 ± 15.2 keV/μm, as previously reported.^30^ Cells were exposed to doses of 0.25, 0.5, 1, 1.5, and 2 Gy, as previously described.^20^ Due to the difference in culture surface, cells grown on Mylar see slightly reduced plating efficiency and survival. To account for this, reference X-ray curves were generated for cells grown on both 6-well plates and Mylar (Supplementary Figure 1). A correction factor of 1.22 was obtained to adjust from doses in plates to doses on Mylar, which was used for alpha particle RBE calculations in Figures 3 and 4, and comparisons with model predictions in Figure 5.

### Biological assays

Overall radiosensitivity was determined using the clonogenic assay, to measure the intrinsic radiosensitivity of cells at different LETs over relevant dose ranges, and fit using the linear-quadratic model.^31^

DNA damage induction and repair was quantified using 53BP1 immunofluorescence, following 2 Gy irradiation, at timepoints ranging from 1 to 24 hours following irradiation in all setups, and fit with a 1-phase exponential decay.

Further details on these assays can be found in the Supplementary Information.

### Medras

Medras is an open-source mechanistic radiation response model. Rather than phenomenological fitting parameters, it models fundamental cellular processes, such as the rate of DNA repair of each pathway and associated likelihood of chromosome aberration formation. This is combined with the mutational profile of cell to predict radiobiological responses. Medras begins with the simulation of radiation-induced DSBs, where their spatial distribution is dependent on the radiation/particle energy, LET, and particle type. The model then calculates the probability of different types of repair and misrepair for each DSB. These rates depend on the spatial distribution of the DSBs, with DSBs in close spatial proximity being more likely to undergo misrepair, as are those which are attempted to be repaired by pathways which are knocked out. Finally, cell fate is determined based on the resulting genetic rearrangements.

Different cell lines are defined in terms of a simplified radiation phenotype, consisting of their genome size, number of chromosomes, activity of key DSB repair and G1 arrest pathways, and cell cycle phase. Detailed information about Medras can be found elsewhere,^26^, ^32^, ^33^ and it is available at https://github.com/sjmcmahon/Medras.

Further developments were introduced to reflect the experiments in this study. Firstly, a new class of DNA repair pathway failure was introduced. Medras modelled pathway function as a binary variable, with pathways either fully functional or fully defective. However, loss of some genes, such as DCLRE1C (Artemis), is known to only affect repair of a subset of complex breaks.^34^ Reflecting this, a new category of pathway defect was introduced, where repair only fails for complex DSBs. This was used to model responses in DCLRE1C- and PRKDC-defective cells. Notably, Medras predicts complex DSBs with a fixed probability reflecting the biological context of the DSB, without any LET dependence. As a result, the relative contribution of these pathways, and all other DSB repair pathways, is also independent of LET on the level of individual breaks.

Similarly, G1 arrest and apoptosis was also previously either fully functional or entirely absent. However, P53 knockout alone had a smaller effect in this cell line, so to reflect this continuous G1 checkpoint activity was used, ranging from 0 (fully absent) to 1 (fully functional), with the fully functional rate using previously-fit values. Finally, it was observed that in these RPE-1 cells with an otherwise normal background, the impact of loss of DNA repair genes was less severe than in some cancer lines. To reflect this, the alt-EJ repair failure probability variable was updated for this experiment.

These 2 quantities were fit to the X-ray response data and then used to predict responses to higher LETs. Supplementary Table 4 shows the parameters used in this study. It should be noted that while these parameters change the quantitative predictions of the model, they do not change the predicted relationship between LET and DNA repair pathways, or the overall ordering of sensitivity between knockouts and LETs.

### Statistical analysis

All X-ray and alpha-particle experiments were performed in triplicate, however, due to beamtime limitations, proton and carbon ion irradiations were performed in duplicate. Unpaired Student *t* test and 1-way analysis of variance were used for statistical evaluation. All statistics and graph plotting used GraphPad Prism 10.0 (GraphPad, USA).

Clonogenic survival data were fit to the linear quadratic equation ($\mathrm{SF}=e^{-(\alpha D+\beta D^{2})}$) using nonlinear regression.^31^ Overall sensitivity was quantified using mean inactivation dose (MID), defined as the area under the dose-response curve for a given condition,^35^ and an exponential interpolation was used to calculate MID for these data (Supplementary Methods). Knockout sensitiser enhancement ratio (SER) values were calculated as the ratio of the MID of wild-type cells to repair-deficient cells and RBE values were calculated as the ratio of the MID of X-ray irradiated to particle irradiated cells (RBE_MID_).

## Results

### Clonogenic survival

**Figure 1:**
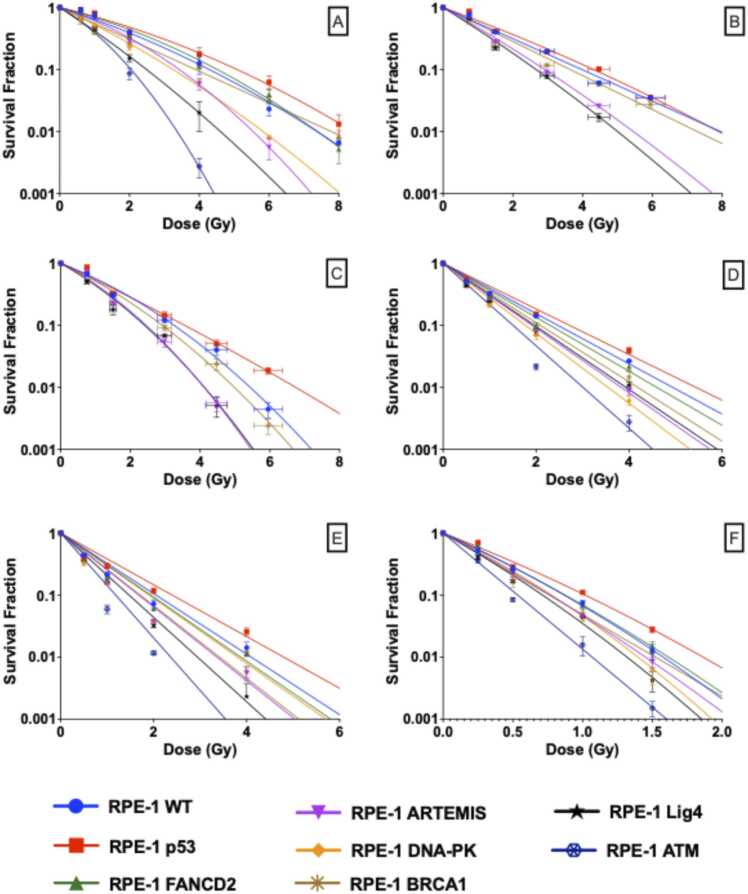
CRISPR-Cas9 genetically modified cell survival curves after exposure to different qualities of radiation: (a) 225 kVp x-rays, (b) 58 MeV protons (LET 1 keV/μm), (c) 11 MeV protons (LET 12 keV/μm), (d) 71/A MeV carbon ions (LET 34 keV/μm), (e) 26/A MeV carbon ions (LET 73 keV/μm), and (f) 2.9 MeV alpha particles (LET 129 keV/μm).

Figure 1 shows the survival curves for each cell model after exposure to X-rays (A), low-LET protons (B), high-LET protons (C), low-LET carbon ions (D), high-LET carbon ions (E), and alpha-particles (F). Across the range of tested LETs, from 0.5 to 129 keV/μm, cellular radiosensitivity follows a similar trend, in descending order of sensitivity: ATM-/-, LIG4-/-, DNA-PK-/-, Artemis-/-, BRCA1-/-, FANCD2-/-, wild-type, p53-/- cells. In other words, ATM null and NHEJ null cells are hypersensitive to all tested radiation qualities. It can also be seen that survival curves demonstrate a more linear and steeper dose-response relationship with increased values of LET.

X-ray, proton, and alpha data adapted from Liberal et al.^20^

### DNA repair

**Figure 2:**
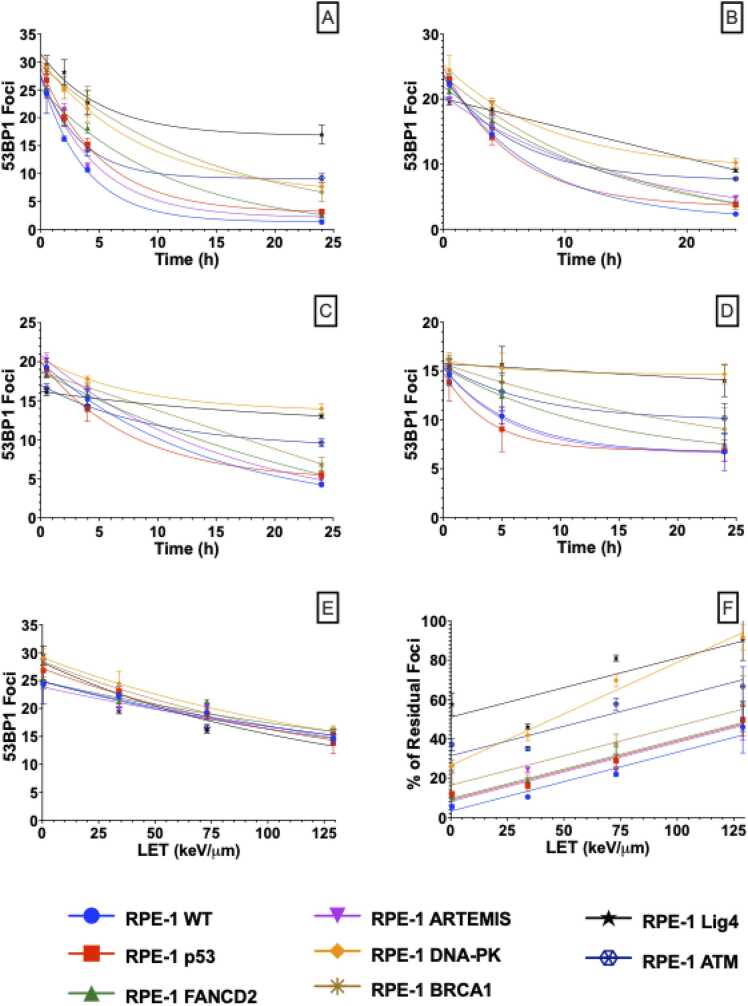
DSB repair kinetics, measured by 53BP1 foci for each cell line after exposure to 2 Gy of different quality radiation as function of time (a) X-rays, (b) 71 MeV Carbon Ions (LET 34 keV/μm), (c) 26 MeV Carbon Ions (LET 73 keV/μm) and (d) 2.9 MeV Alpha Particles (LET 129 keV/μm). (e) Number of induced foci at 1 h as a function of LET. (f) Percentage of induced foci remaining 24 h after exposure as a function LET.

Figure 2 shows the repair kinetics of 53BP1, a DSB marker, after exposure to 2 Gy of X-rays (A), low-LET carbon ions (B), high-LET carbon ions (C) and alpha-particles (D) as a function of time from 1 to 24 h after irradiation (Supplementary Table 1). Across all tested LETs, mutations in key DSB repair genes have a dramatic impact on the repair kinetics and capability to clear foci (Figure 2A-D). Proteins associated with the NHEJ repair pathway such as Lig4, DNA-PK and DNA damage sensor ATM have the greatest impact on the clearance of foci, even for high-LET radiation (Figure 2E and F).

X- ray and alpha data adapted from Liberal et al.^20^

The initial number of foci also decreases with LET (Figure 2E). For RPE-1 wild-type cells, 2 Gy of X-rays induces 24.3 ± 0.2 53BP1 foci 1 h after exposure, followed by 22.4 ± 0.2 with low LET Carbon ions, 19.3 ± 1.2 by high-LET Carbon ions and finally 14.6 ± 0.9 with alpha-particles, which is believed to reflect clustering of multiple DSBs within single tracks. High-LET induced foci also tend to be larger and brighter, as seen in previous studies,^36^, ^37^, ^38^ have slower repair kinetics, and a higher proportion of persistent foci (Figure 2F), with an approximately linear dependence of residual foci on LET. The percentage of repair after 24 h in RPE-1 wild-type cells is 94.3 ± 1.7, 89.5 ± 0.6, 78.0 ± 0.9, and 50.1 ± 12.3 for X-ray, low- and high-LET carbon ions, and alpha-particles respectively. Interestingly, the slope of the relationship between percentage persistent DSBs and LET is similar across most gene knockouts lines (*P* > .46), with the exception of DNA-PK null cells (*P* = .038), which exhibit a notably steeper increase in unrepaired DSBs with increasing LET. This suggests that while the impact of DNA repair deficiencies on DSB clearance is generally consistent across different LETs, DNA-PKcs may play a particularly crucial role in processing complex DNA damage induced by higher LETs. However, it is important to note that particle-induced DSB repair kinetic parameters should be treated with caution due to their limited number of time-points.

### Correlation of RBE and SER with LET of the incident radiation

**Figure 3:**
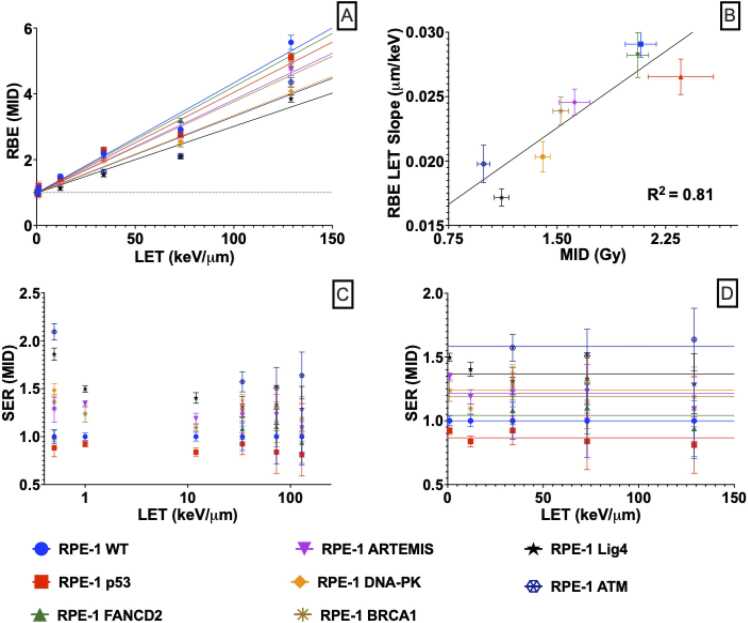
(a) Correlation between RBE_MID_ and LET. (b) Correlation between RBE-LET fit slope and X-ray MID of each cell line. (c) SER values obtained for each protein in the different irradiation scenarios. (d) Correlation between Sensitiser Enhancement Ratio (SER) and LET of particle radiation, excluding X-ray SER values.

High-LET radiation kills the same number of cells with lower doses than X-rays, giving high RBE values. RPE-1 wild-type cells showed RBE_MID_ values of 1.16 ± 0.07, 1.49 ± 0.06, 2.18 ± 0.11, 2.92 ± 0.21 and 5.56 ± 0.21, for low- and high-LET protons, low- and high-LET carbon ions and alpha-particles, respectively (Supplementary Table 2). A trend of approximately linearly increasing RBE_MID_ is seen for all analysed knockout lines (Figure 3A). Interestingly, the RBE-LET slope for each cell line was seen to be linearly related to the X-ray radiosensitivity measured by MID (R^2^ = 0.80) (Figure 3B), showing that more radioresistant cells tend to see greater RBEs, as supported by other models,^19^, ^39^ but again not indicating any other dependence on particular repair pathways. A version of Figure 3A without correction for the Mylar growth conditions is presented in Supplementary Figure 2.

X-ray, proton, and alpha data adapted from Liberal et al.^20^

We also evaluated Sensitiser Enhancement Ratio (SER), to study the importance of a particular protein between exposures in the same irradiation scenario. Figure 3C shows 3 clusters of proteins: cluster 1, ATM and LIG4 that play a crucial role in X-ray response with SER values of 2.09 ± 0.17 and 1.88 ± 0.14, respectively, followed by cluster 2, DNA-PK, BRCA1 and Artemis with SER values of 1.48 ± 0.11, 1.37 ± 0.09, and 1.28 ± 0.12, proteins which have a moderate impact in radiation response and finally cluster 3, FANCD2 and P53, proteins that have no impact or a protective effect after X-ray exposures (Supplementary Table 3). For all knockouts the SER is somewhat lower for particle exposures than X-ray exposures, although the ordering of SERs remains unchanged (1-way analysis of variance tests for SER variation with LET gave *p*-values of *P* = .035 and *P* = .085 for the data set with and without X-rays data, respectively). However, there was no significant slope with LET for charged particles (Figure 3D, *P* > .12 for SER vs LET slope >0), suggesting again that there was no significant difference in DNA repair pathway dependence as a function of particle LET.

### Mechanistic modelling of radiation effects in cell survival

**Figure 4:**
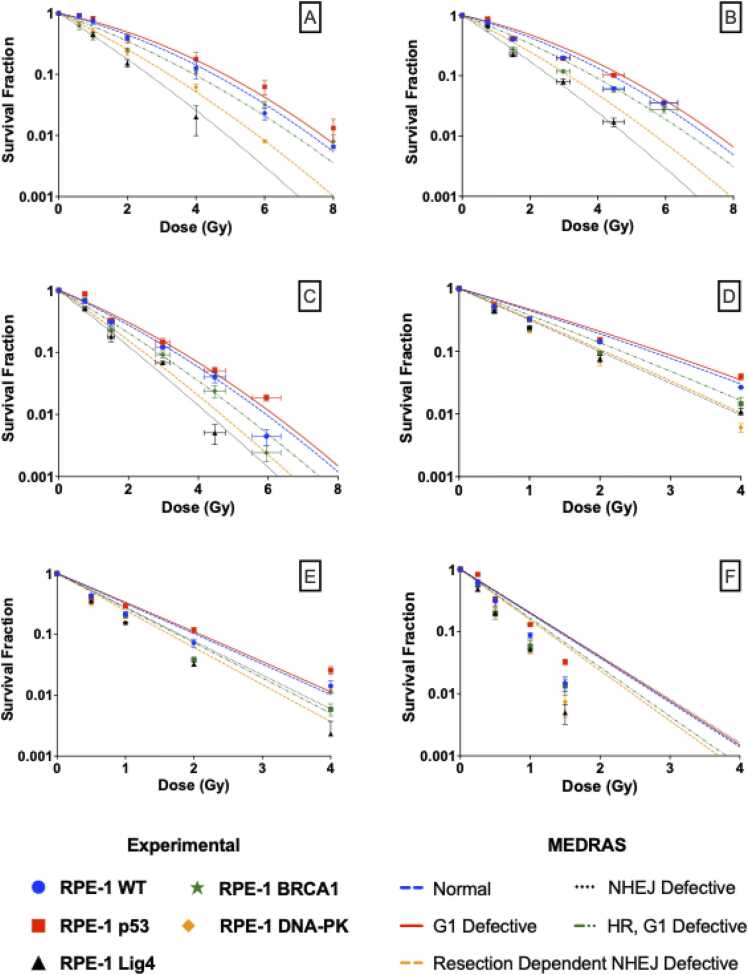
Medras cell survival curves for each cell model and its comparison with experimental data: (a) 225 kVp X-rays, (b) 58 MeV Proton (LET 1 keV/μm), (c) 11 MeV Proton (LET 12 keV/μm), (d) 71 MeV/A Carbon Ions (LET 34 keV/μm), (e) 26 MeV/A Carbon Ions (LET 73 keV/μm) and (f) 2.9 MeV alpha particles (LET 129 keV/μm).

Figure 4 shows the survival curves produced by Medras. Medras can effectively reproduce the response of wild-type cells with no cell-specific fitting, and captures the relative impacts of a range of different DNA repair defects. For high-LET particles, Medras also reproduces the overall trends in RBE_MID_ as a function of LET, in good quantitative agreement with proton and carbon ion exposures. However, while predicting the highest RBE for alpha particles, it significantly under-estimates the overall magnitude of response (Figure 4F). The poor predictive performance on alpha-particle exposures can be in part explained by their distinct irradiation setup on Mylar (Supplementary Figure 1).

**Figure 5:**
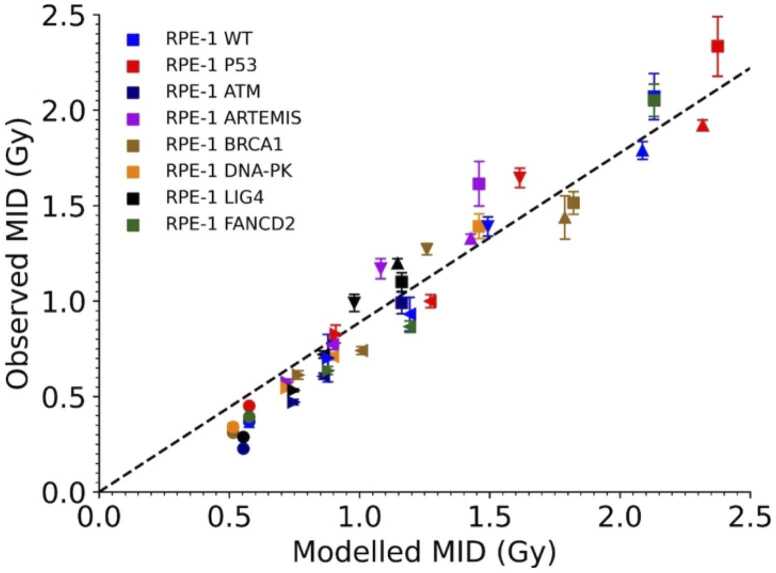
Medras accurately predicts radiation response in different cell models, shown by the correlation between Medras-predicted and experimentally obtained MID values. Predicted alpha MID values were corrected based on estimate of impact on cell viability of irradiation on Mylar. Cell lines are coloured as in other figures, with symbol representing radiation type (Square: X-ray; Upwards and downwards triangles: low and high-LET protons; Left and right triangles: low and high-LET carbon ions; Circles: alpha particles).

Figure 5 presents a correlation of Medras-predicted and experimentally observed MID, for all cell models and radiation qualities. A good correlation is seen, with the best-fitting slope coefficient of 0.89 ± 0.02 and a correlation coefficient of R^2^ = 0.93. This suggests that, in addition to capturing the impact of DNA repair defects on responses to X-rays, Medras can also predict their impact on responses to high-LET exposures without any additional fitting and, of relevance to this work, without assuming a change in repair pathway dependence with LET. A version of Figure 5 without correction for the Mylar growth conditions is presented in Supplementary Figure 3.

## Discussion

Radiotherapy has seen remarkable technical advancements enabling a high degree of anatomical personalisation. Despite these advancements, radiotherapy often lacks biological personalisation, with most patients receiving a ‘one size fits all’ treatment. This approach is an oversimplification, as extensive evidence indicates that cancers of the same type can exhibit vastly different radiosensitivity.^25^, ^27^ One of the crucial determinants of cancer radiosensitivity is DNA damage signalling and repair pathway activity. Specific genes whose deletion is associated with loss of DNA repair function, such as BRCA1, ATM, DNA-PKcs, P53, are known to affect cellular radiosensitivity. However, direct measurement of intrinsic radiosensitivity remains challenging in the clinic. This complexity is further compounded by the increasing availability of advanced radiotherapy techniques, such as protons and carbon ions, where dose-response relationships differ from those of X-rays. Accurate mechanistic models of radiation response offer an appealing approach to these problems.

However, developing a predictive model for biological responses to ionising radiation is challenging, particularly when comparing X-rays with particle radiation. Difficulty arises from their distinct ionisation patterns and uncertainty surrounding their RBE across different biological systems. Despite growing knowledge of the radiobiology of high-LET radiation, uncertainties regarding RBE^40^ impact the prediction of the clinical benefit of transitioning patients from standard X-ray treatment to these more expensive and less widely available facilities.

We applied CRISPR/Cas9 technology to knock out key genes involved in different DSB repair pathways in RPE1 cells, and irradiated them with different radiation qualities, ranging from X-rays to alpha-particles. The findings of this study emphasise the significant impact of LET and DNA repair mechanisms on cellular radiosensitivity, and help to elucidate the differential response of cells to various forms of radiation.

The clonogenic survival data reveal a clear trend: increased LET correlates with heightened cellular radiosensitivity across all tested cell lines. This observation aligns with existing literature, confirming that high-LET radiation is more effective at inducing cell death than low-LET radiation.^7^ The ranking of cell sensitivity, from most to least sensitive, remains consistent across all LET values: ATM-/-, LIG4-/-, DNA-PKcs-/-, Artemis-/-, BRCA1-/-, FANCD2-/-, wild-type, and P53-/-. This hierarchy highlights the critical roles of NHEJ and ATM dependent pathways in mediating the cellular response to radiation-induced DNA damage, in agreement with previously publications.^21^, ^41^ The SER for all gene defects was lower for charged particle exposures compared to X-rays. However, this reduction in SER is not a continuous, LET-dependent decline. Rather, it reflects an initial drop in SER between X-rays and the lowest LET charged particles, after which no significant trend is observed. Therefore, the primary difference appears to lie between X-rays and particle therapy, rather than among particles of increasing LET. Interestingly, the greatest drop in SER was associated with most sensitising gene defects (ATM and Lig4), which gives the appearance of a relatively greater contribution of HR at high LETs, if the changes in HR and NHEJ SERs with LET are directly compared.^18^ However, it is also consistent with an “overkill” effect where it is more difficult to further sensitise the already hyper-sensitive NHEJ-defective cells, as seen in our RBE data.

The analysis of 53BP1 foci dynamics provides further insight into the differential DNA repair capacities. The number of foci induced fell with increasing LET, with high-LET radiation producing fewer visible foci but more complex DNA lesions that were slower to clear. These factors could be explained by clustered DSBs around tracks at higher LET, which cannot be optically resolved. Proteins involved in NHEJ, particularly Lig4, DNA-PK, and ATM, were crucial for efficient repair, even with high-LET radiation. By contrast, while loss of HR genes slightly slowed repair in all cases, this was consistently less impactful than NHEJ, and did not increase in importance for charged particles. These data demonstrate a moderate correlation between MID and residual damage across the tested cell models (R^2^ = 0.58, Supplementary Figure 4), consistent with previous findings reported in a broader range of cell lines.^27^ Moreover, the significance of the correlation increases when p53 null cells are excluded from analyses R^2^ = 0.66, highlighting a correlation between residual damage and intrinsic radiosensitivity. Observations from this study differ from some previous reports. Fontana et al. demonstrated that inhibition of HR strongly delayed DSB repair and reduced cell survival after exposure to protons, but had minimal impact after photon exposure, suggesting a preferential requirement of HR for the repair of higher-LET-induced damage.^15^ Other studies have presented similar observations, although with varying degrees of impact assigned to HR loss in charged particle therapy.^14^, ^15^, ^16^, ^17^, ^42^, ^43^

In contrast, our findings support the view that NHEJ repair is the most critical pathway for cell survival, and that HR’s role is less significant, even under high-LET conditions. This interpretation is supported by our mechanistic modelling. Medras effectively predicted cell survival across a range of LETs and genetic backgrounds, with strong correlation between experimental and model-predicted MIDs (R² = 0.90), but without any assumption of changing complexity of individual DSBs or pathway dependence with LET. This is achieved without knockout- or LET-specific fitting to this data. Similar findings have been reported in a number of studies, which suggest NHEJ’s continuing importance and a lack of elevated HR-dependence at higher LETs.^20^, ^21^, ^44^, ^45^, ^46^, ^47^ In this cell model, Medras slightly under-estimates overall sensitivity, which is more pronounced at higher LETs (slope coefficient 0.88). This may reflect that not all parameters which affect RBE are fully taken into account in this model, such as differences in nuclear geometry which play an increasingly significant role at higher LETs, but does not affect the predictions of SER or the broader relationship between DNA repair pathways.

The mechanisms by which DNA repair pathways are selected by cells remain unclear, with evidence that it is a multivariable process dependent on factors including incident radiation quality, cell cycle stage, and dose.^48^ Notably, these data suggest an increasing importance for alt-EJ pathways at high-LET, to repair complex damage. Alt-EJ is often viewed as a fallback mechanism, but also has its own genetic dependencies. Complex or clustered DNA damage disrupts the balance among repair pathways, leading to an enhanced G2 checkpoint arrest primarily regulated by ATR, increased DNA end-resection, and a more prominent role for proteins such as DNA-PKcs, Artemis, and FANCD2.^23^, ^48^, ^49^, ^50^ Moreover, observed distinct relative importances and biological effects of DNA-PKcs and LIG4, despite both acting within the canonical NHEJ pathway, reflects their nonoverlapping roles in DSB sensing/processing and end ligation, respectively, which highlights the need to consider individual protein functions rather than relying solely on pathway classification.

While our study does not explore the interplay between these pathways, it enables the isolation of individual pathway effects to better understand their relative contributions, suggesting their role is not strongly LET-dependent in this system. Further studies using cells with diverse genetic backgrounds are needed to resolve ambiguities surrounding interactions between DNA repair pathways and the role of sublethal damage in radiation response.

A limitation of this study is that it relied primarily on immortalised RPE-1 cells with specific CRISPR/Cas9-mediated gene knockouts, which, while allowing controlled investigation of individual DNA repair pathways, may not fully replicate the complex genetic, epigenetic, and microenvironmental factors influencing DNA repair and radiosensitivity in diverse cancers. Furthermore, although Medras accurately predicted trends without cell-specific fitting, it remains a simplified representation of cellular processes, and additional validation across a wider range of genetic backgrounds and radiation conditions will be necessary to confirm its generalisability.

Resolution of these questions would have significant implications for the future of personalised radiotherapy. The demonstrated variability in radiosensitivity due to genetic differences underscores the potential benefits of incorporating genetic profiling into clinical decision-making, supported by suitable modelling, which may inform both selection between conventional X-ray or particle therapy, as well as overall dosimetric decisions, to maximise therapeutic outcomes while minimising side effects.

## Conclusion

The mechanisms by which DNA repair pathways are selected after exposure to different radiation qualities remains unclear. Despite extensive radiobiological data, this critical issue needs to be thoroughly investigated both in vitro and in vivo to fully optimise radiotherapy. This study underscores the critical role of DNA repair mechanisms in radiotherapy. Conducting similar studies with diverse genetic backgrounds will be essential to resolving ambiguities regarding DNA repair pathways following high-LET exposures. The responses of genetically modified cell lines to varying LETs highlight the complex relationship between radiation quality and DNA damage repair. Future research should focus on refining these models and expanding our knowledge of the genetic factors influencing radiosensitivity to fully harness the potential of advanced radiotherapy techniques.

## Funding

UKRI Future Leaders Fellowship MR/T021721/1 (SJM).

Carbon ion beam time was obtained under the experiment nº P1363-H of the iPAC 2023 call.

## Declaration of Competing Interest

The authors declare the following financial interests/personal relationships which may be considered as potential competing interests: Stephen McMahon reports financial support was provided by UK Research and Innovation. If there are other authors, they declare that they have no known competing financial interests or personal relationships that could have appeared to influence the work reported in this paper.

## Footnotes

Appendix A Supplementary data associated with this article can be found in the online version at doi:10.1016/j.ijpt.2025.101198.
