# Supplementary material for: Assessing the Relative Contribution of DSB Repair Proteins as a Function of LET
Source: Int J Part Ther. 2025 Jul 26;17:101198. doi: 10.1016/j.ijpt.2025.101198 (PMC12356031; doi:10.1016/j.ijpt.2025.101198)
Supplement: Supplementary file 1 — Supplementary material [file mmc1.pdf]

Assessing the relative contribution of various DSB repair proteins as a function of LET

Francisco D.C. Guerra Liberal [1], Shannon Thompson [1], Lydia Gardner [1], Jason L. Parsons [2], François Chevalier[3], Kevin Tabury[4], Stephen J. McMahon [1]

1 - The Patrick G Johnston Centre for Cancer Research, Queen's University Belfast 2 - Institute of Cancer and Genomic Sciences, University of Birmingham
3- CIMAP, Université de Caen Normandie

Supplementary Information

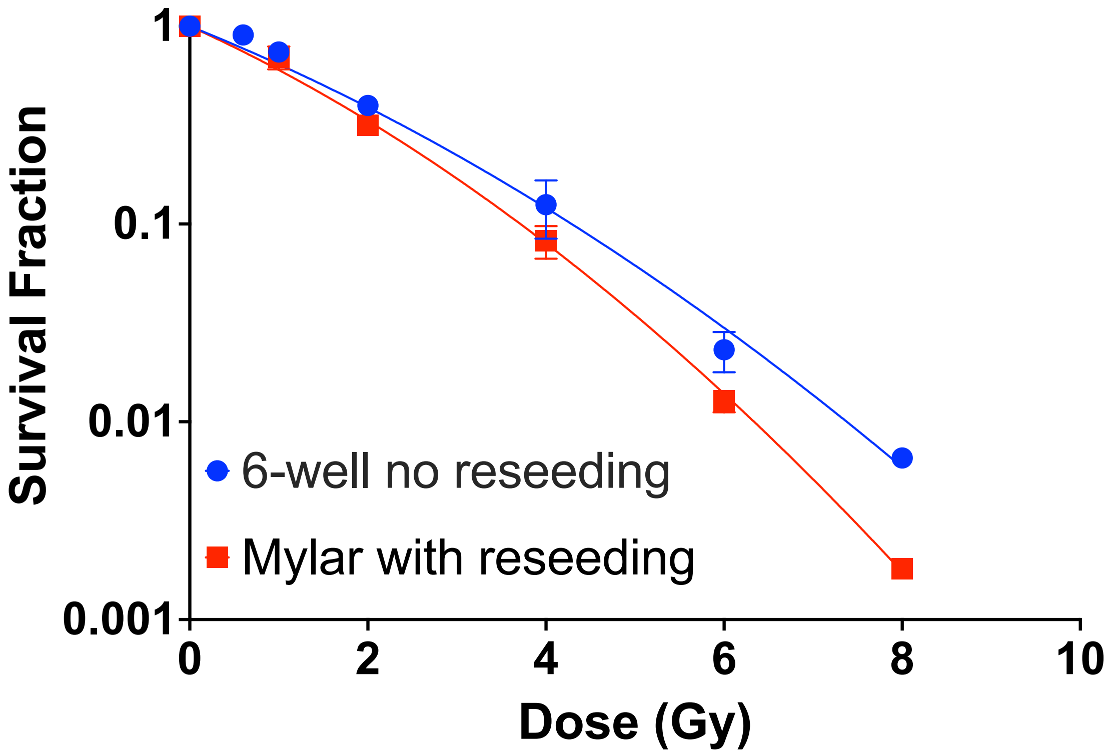

Supplementary Figure 1: Survival curves of RPE-1 wild-type cells exposed to X-rays in two different setups: (blue) irradiation in 6-well plates and no reseeding, (red) irradiation in Mylar dishes with reseeding to 6-well plates. Reduction in viability is equivalent to a factor of 1.2 change in the dose delivered.

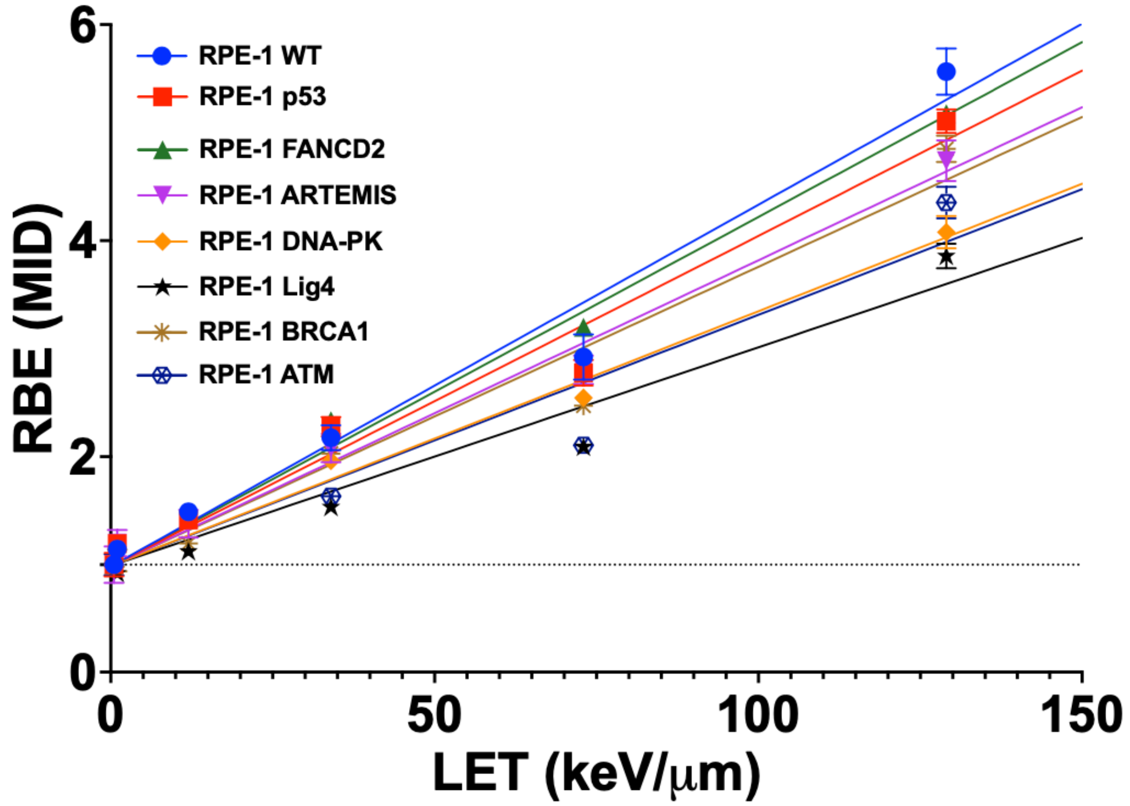

Supplementary Figure 2: Correlation between RBE_{MID} and LET, plotted as in Figure 3 (a) in the main text, with predicted alpha MID values not corrected based on the estimate of impact on cell viability of irradiation on Mylar.

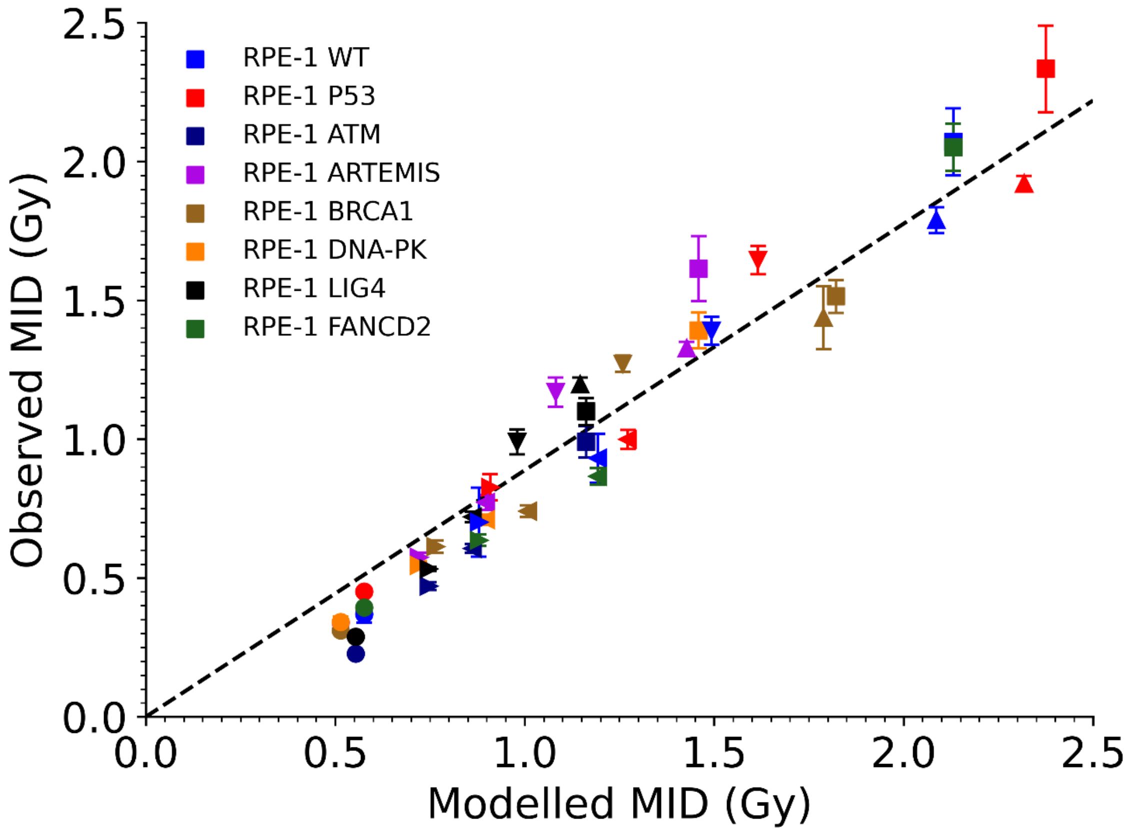

Supplementary Figure 3: Medras model prediction compared to experimental observation, plotted as in Figure 5 in the main text, with predicted alpha MID values not corrected based on the estimate of impact on cell viability of irradiation on Mylar, with a new slope coefficient of 0.88±0.04, and R^2=0.90.

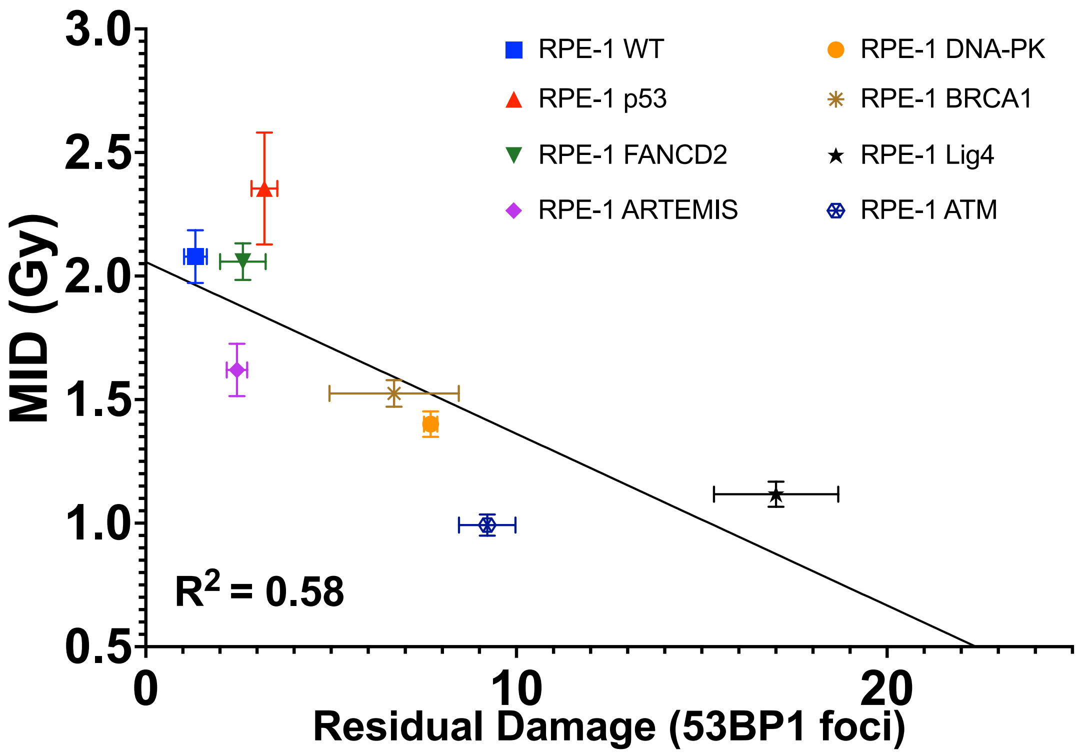

Supplementary Figure 4: Correlation between MID for each cell model when exposed to X-rays and residual damage, 24 hours after exposure to 2 Gy of X-rays.

Supplementary Table 1: 53BP1 Foci for different cell lines exposure to 2 Gy of different radiation times and fixed at 1, 2, 4 and 24 Hours, data presented as mean and standard deviation

Cell Line	**Radiation Type**	**1 Hour**	**2 Hours**	**4 Hours**	**24Hours**
RPE1 WT	X-ray	24.3 ± 0.2	16.2 ± 0.5	10.7 ± 0.4	1.3 ± 0.3
	Carbon Ion 71 MeV	22.4 ± 0.1		14.6 ± 0.3	2.4 ± 0.1
	Carbon Ion 26 MeV	19.3 ± 1.2		15.2 ± 1.2	4.2 ± 0.2
	Alpha Particles	14.6 ± 1.0		15.3 ± 1.1	6.7 ± 1.9
RPE1 P53	X-ray	26.8 ± 1.6	20.1 ± 1.5	15.3 ± 1.1	3.2 ± 0.3
	Carbon Ion 71 MeV	23.2 ± 0.2		14.1 ± 1.1	3.8 ± 0.4
	Carbon Ion 26 MeV	19.0 ± 0.9		13.9 ± 1.5	5.5 ± 0.6
	Alpha Particles	13.9 ± 1.9		9.1 ± 2.3	6.9 ± 1.1
RPE1 ATM	X-ray	24.8 ± 4.0	19.9 ± 1.5	14.2 ± 1.1	9.2 ± 0.8
	Carbon Ion 71 MeV	22.1 ± 1.8		15.6 ± 0.8	7.8 ± 0.2
	Carbon Ion 26 MeV	16.6 ± 0.5		14.3 ± 0.2	9.6 ± 0.5
	Alpha Particles	15.3 ± 0.9		12.9 ± 2.0	10.2 ± 1.5
RPE1 Artemis	X-ray	23.9 ± 0.5	21.5 ± 1.1	11.3 ± 0.7	2.5 ± 0.3
	Carbon Ion 71 MeV	19.9 ± 0.2		15.8 ± 1.9	4.8 ± 0.3
	Carbon Ion 26 MeV	20.2 ± 0.9		16.3 ± 0.2	4.8 ± 0.3
	Alpha Particles	14.7 ± 0.7		10.2 ± 0.6	6.7 ± 0.8

	X-ray	28.8 ± 1.1	25.4 ± 2.0	23.0 ± 2.7	6.7 ± 1.7
	Carbon Ion 71 MeV	22.9 ± 0.2		17.9 ± 0.2	4.0 ± 1.0
RPE1 BRCA1	Carbon Ion 26 MeV	18.3 ± 0.4		16.9 ± 0.4	6.9 ± 0.9
	Alpha Particles	15.5 ± 0.8		13.9 ± 1.7	9.1 ± 2.1
	X-ray	29.1 ± 1.5	25.1 ± 0.4	21.7 ± 2.4	7.7 ± 0.2
	Carbon Ion 71 MeV	24.4 ± 2.2		19.4 ± 0.7	10.3 ± 0.7
RPE1 DNA-PK	Carbon Ion 26 MeV	20.0 ± 0.7		17.8 ± 0.4	14.0 ± 0.7
	Alpha Particles	16.0 ± 0.9		15.3 ± 1.5	14.7 ± 1.0
	X-ray	29.5 ± 1.6	28.2 ± 2.2	22.7 ± 2.1	17.0 ± 1.7
	Carbon Ion 71 MeV	19.6 ± 0.5		18.5 ± 1.2	9.1 ± 0.3
RPE1 LIG4	Carbon Ion 26 MeV	16.1 ± 0.5		15.4 ± 1.0	13.1 ± 0.3
	Alpha Particles	15.5 ± 0.8		15.7 ± 1.8	14.0 ± 1.6
	X-ray	24.7 ± 1.0	19.7 ± 1.0	18.2 ± 0.9	2.6 ± 0.6
	Carbon Ion 71 MeV	21.2 ± 0.4		16.7 ± 0.4	4.0 ± 0.3
RPE1 FANCD2	Carbon Ion 26 MeV	18.3 ± 0.3		15.8 ± 0.1	5.6 ± 1.1
	Alpha Particles	15.1 ± 1.6		12.5 ± 2.1	7.5 ± 1.0

Supplementary Table 2: RBE Values for different cell models exposed to Proton, Carbon Ions and Alpha particles presented as mean and standard deviation.

Cell Line	**Proton 58 MeV**	**Proton 11 MeV**	**Carbon Ion 71 MeV**	**Carbon Ion 26 MeV**	**Alpha Particles**
RPE1 WT	1.16 ± 0.07	1.49 ± 0.06	2.18 ± 0.11	2.92 ± 0.21	5.56 ± 0.21
RPE1 P53	1.19 ± 0.08	1.41 ± 0.08	2.28 ± 0.09	2.78 ± 0.12	5.11 ± 0.11
RPE1 ATM			1.63 ± 0.07	2.11 ± 0.09	4.35 ± 0.15
RPE1 Artemis	1.20 ± 0.12	1.38 ± 0.13	2.08 ± 0.13	2.08 ± 0.13	4.74 ± 0.19
RPE1 BRCA1	1.04 ± 0.10	1.20 ± 0.06	2.03 ± 0.07	2.47 ± 0.08	4.85 ± 0.12
RPE1 DNA-PK			1.96 ± 0.06	2.54 ± 0.07	4.08 ± 0.15
RPE1 LIG4	0.92 ± 0.04	1.12 ± 0.06	1.53 ± 0.05	2.09 ± 0.04	3.86 ± 0.11
RPE1 FANCD2			2.33 ± 0.05	3.20 ± 0.05	5.17 ± 0.07

Supplementary Table 3: SER Values for different cell models exposed to Proton, Carbon Ions and Alpha particles presented as mean and standard deviation.

Cell Line	**X-ray**	**Proton 58 MeV**	**Proton 11 MeV**	**Carbon Ion 71 MeV**	**Carbon Ion 26 MeV**	**Alpha Particles**
RPE1 P53	0.88 ± 0.09	0.92 ± 0.03	0.84 ± 0.04	0.92 ± 0.11	0.84 ± 0.22	0.81 ± 0.22
RPE1 ATM	2.09 ± 0.09			1.57 ± 0.10	1.51 ± 0.21	1.64 ± 25.15
RPE1 Artemis	1.28 ± 0.13	1.34 ± 0.03	1.89 ± 0.06	1.23 ± 0.12	1.23 ± 0.21	1.09 ± 0.25
RPE1 BRCA1	1.36 ± 0.08	1.24 ± 0.09	1.10 ± 0.04	1.27 ± 0.11	1.15 ± 0.21	1.19 ± 0.24
RPE1 DNA-PK	1.48 ± 0.07			1.34 ± 0.11	1.29 ± 0.21	1.08 ± 0.25
RPE1 LIG4	1.86 ± 0.06	1.50 ± 0.03	1.40 ± 0.06	1.31 ± 0.11	1.33 ± 0.20	1.29 ± 0.23
RPE1 FANCD2	1.01 ± 0.06			1.08 ± 0.11	1.10 ± 0.21	0.94 ± 0.22

Supplementary Methods

Clonogenic cell survival

Cells were seeded the day before irradiation with an optimised cell density according to the dose and radiation type. After irradiation, cells were incubated for 7 days before staining. The colonies were then stained with a 4% crystal violet solution in ethanol and were manually counted, with a colony defined as consisting of at least 50 cells. From these counts, plating efficiency (PE) and survival fraction (SF) were calculated. SF was determined by the number of colonies formed after irradiation divided by the number of cells seeded, corrected for the PE of unirradiated cells.

DNA damage by immunofluorescence assay

Following 2 Gy irradiation, cells were fixed in a 50:50 methanol-acetone solution and permeabilized (0.5% Triton X-100 in PBS) at predetermined time points before being blocked in blocking buffer (5% FBS and 0.1% Triton X-100 in PBS) and stained with 53BP1 primary antibody (1:5000) (#NB100-304, Novus Biologicals, USA) for 1 h. Cells were then washed three times in PBS and stained with Alexa Flour 568 goat anti-rabbit IgG secondary antibody (#A21429, Life Technologies, USA) (1:2000) in the dark for 1h. Following staining, the cells were washed three times in PBS and mounted onto microscope slides using Prolong Gold anti-fade reagent with DAPI (#P36930, Invitrogen, USA). Foci were manually counted from the whole nucleus of 50 randomly selected cells on each sample with a Nikon Eclipse Ti microscope (Nikon Corporation, Japan), using a 60x objective.

Data are presented as the mean values of foci per cell and the respective standard deviation of three independent experiments. Data presented here is corrected for background damage levels by subtracting the number of foci in unirradiated cells. For repair kinetic analysis, foci data were then fit with an exponential decay in GraphPad Prism 10.0, $N = (N_0 - plateau) * e^{-kt} + plateau$, where N_0 represents the initial number of foci, plateau represents the residual damage and k is the rate of DSB repair. Yields of initial foci as a function of LET were fit by an equation of the form $N = \frac{N_0}{k\,LET}(1 - e^{-k\,LET})$, where N_0 is the number of foci at low LET, and k is a scaling constant describing the clustering of foci around tracks as a function of LET (see section bellow for further details).

Medras Parameters

Supplementary Table 4: Medras Model parameters used in this study

Standard Parameters	Interpretation	Value
**σ**	Rejoining range	0.0418±0.0003 R
**μ_{NHEJ}**	NHEJ misrepair probability	0.985±0.001
**λ_m**	Complex break probability	0.43±0.02
**p_{fail}**	Repair failure probability	0.74±0.09
**λ_f**	Fast repair Rate	2.1±0.2 h^{-1}
**λ_s**	Slow repair Rate	0.26±0.02 h^{-1}
**λ_m**	Alt-EJ repair rate	0.0085±0.001 h^{-1}
**R**	Nucleus radius	4.2±0.4 µm

Updated Parameters	Interpretation	Value
**μ_{MMEJ}**	Alt-EJ Misrepair probability	0.84±0.03
**$p_{G1Arrest}$**	G1 Arrest Failure in RPE-1 P53 KO	0.73±0.11

MID calculation

Mean Inactivation Doses (MID) were calculated based on the area under the experimental dose-response curves. This was calculated using an exponential extrapolation between points, rather than a linear joining, as the latter approach can significantly over-estimate the area under the curve between two points in an exponential dose response when survival changes significantly between two dose points.

The curve between two points was then modelled as:

$$S_{n+1} = S_n e^{-k(D_{n+1}-D_n)}$$

Where S_n is the survival at the n-th dose point, with dose D_n. k is then a slope parameter describing how quickly cells are killed in that dose step, which is given by:

$$k = \frac{-\ln \frac{S_{n+1}}{S_n}}{D_{n+1} - D_n}$$

The area in each dose step is then given by integrating survival between these two points, given as:

$$\int_{D_n}^{D_{n+1}} S \, dD = \int_0^{\Delta D} S_n e^{-kD} \, dD = \frac{S_n}{k}\left(1 - e^{-k\,\Delta D}\right)$$

Where $\Delta D = D_{n+1} - D_n$ is the size of the dose step between measurements n and $n+1$. This quantity is then summed for all measured points in the dose response curve to give an area under the measured curve.

Finally, the area of the 'tail' of the dose response curve beyond the last measured point is estimated by a simple exponential extrapolation from the final measured point using the slope in the final dose step, giving an area of $\frac{S_N}{k_N}$, where S_N is the final measured survival value and k_N is the calculated slope coefficient for the final dose step. This is a small contribution to MID, typically less than 1%.

Induced 53BP1 foci fit

As described in the main text, the number of 53BP1 foci observed fell with increasing LET. This was believed to be the result of DSBs clustering around tracks forming clusters of 53BP1 foci which cannot be resolved. To test this, we applied a simple model of DSB clustering.

We assumed that the number of DSBs induced per track is, on average, proportional to the LET:

$$N_{DSB/Track} = \lambda \, LET$$

The number of tracks per cell is then given by:

$$N_{Track} = \frac{N_{DSB}}{\lambda \, LET}$$

We then assume that, due to resolution limits, we can then only detect tracks with foci, but not distinguish the number of foci per track. The number of observed foci is then the number of tracks incident on the cell which induced one or more DSB.

As the average number of DSBs per track is $\lambda \, LET$, if we assume that these DSBs are Poisson distributed, then the number of tracks with zero damage events is $e^{-\lambda \, LET}$, so the number of tracks with at least one DSB, and thus number of foci, is:

$$N_{foci} = N_{Track}\left(1 - e^{-\lambda \, LET}\right) = \frac{N_{DSB}}{\lambda \, LET}\left(1 - e^{-\lambda \, LET}\right)$$

This function was then used to describe the number of induced foci observed, by fitting N_{DSB} (equivalent to the foci count at low LET), and λ, describing how DSBs cluster around the tracks.
